# Medicaid Expansions: Probing Medicaid’s Filling of the Cancer Genetic Testing and Screening Space

**DOI:** 10.3390/healthcare10061066

**Published:** 2022-06-08

**Authors:** Stephen M. Modell, Lisa Schlager, Caitlin G. Allen, Gail Marcus

**Affiliations:** 1 Epidemiology, Center for Public Health and Community Genomics, School of Public Health, University of Michigan, M5409 SPH II, 1415 Washington Heights, Ann Arbor, MI 48109, USA; 2 Public Policy, FORCE: Facing Our Risk of Cancer Empowered, 16057 Tampa Palms Boulevard W, PMB #373, Tampa, FL 33647, USA; lisas@facingourrisk.org; 3 Department of Public Health Sciences, College of Medicine, Medical University of South Carolina, 22 Westedge, Room 213, Charleston, SC 29403, USA; allencat@musc.edu; 4 Genetics and Newborn Screening Unit, North Carolina Department of Health and Human Services, C/O CDSA of the Cape Fear, 3311 Burnt Mill Drive, Wilmington, NC 28403, USA; gail.marcus@dhhs.nc.gov

**Keywords:** Medicaid, African Americans, Latinos, rural population, breast cancer, colorectal cancer, cancer screening, genetic testing

## Abstract

Cancer is the third largest source of spending for Medicaid in the United States. A working group of the American Public Health Association Genomics Forum Policy Committee reviewed 133/149 pieces of literature addressing the impact of Medicaid expansion on cancer screening and genetic testing in underserved groups and the general population. Breast and colorectal cancer screening rates improved during very early Medicaid expansion but displayed mixed improvement thereafter. Breast cancer screening rates have remained steady for Latina Medicaid enrollees; colorectal cancer screening rates have improved for African Americans. Urban areas have benefited more than rural. State programs increasingly cover *BRCA1/2* and Lynch syndrome genetic testing, though testing remains underutilized in racial and ethnic groups. While increased federal matching could incentivize more states to engage in Medicaid expansion, steps need to be taken to ensure that they have an adequate distribution of resources to increase screening and testing utilization.

## 1. Introduction: Medicaid Expansion and Cancer

Medicaid has been an essential pillar of healthcare support for lower-income families in the United States since 1965. It currently serves over 75 million people, comprising children (37.5% of recipients), adults, individuals newly eligible under Medicaid expansion, the disabled, and the elderly [1]. As a program defining healthcare, it is more of a living, breathing entity than a static facilitator of healthcare. In other countries, such as Ireland, Cyprus, Thailand, and South Africa, where the public system serves lower-income families and those with complex diseases, the trend is toward more universal healthcare [2]. At home, Medicaid’s focus has shifted over time, from health insurance for those depending on federal cash subsidies for housing, to a source of supplemental or extended medical coverage for maternity and newborn care and persons with kidney failure, to an important resource for the elderly and disabled [3]. The Patient Protection and Affordable Care Act of 2010, P.L. 111–148 (ACA) [4], expanded Medicaid coverage to adults with incomes up to 133% of the Federal Poverty Level, effectively 138%, given that Medicaid expansion allows 5% of income to be ignored (FPL—USD 17,609 for an individual in 2020) [5]. Enrollment in Medicaid increases during periods of economic downturn, such as the COVID-19 pandemic, when it took on another 5.3 million individuals [3].

The timeline for the additional Medicaid enrollments occurring as a result of the ACA’s enactment bears consideration for the tracking of any services guaranteed at no cost under the ACA. In July 2012, an amendment was made to Oregon’s Medicaid waiver to reduce spending growth and improve quality and access by enrolling members in locally governed coordinated care organizations [6]. In January 2013, states were allowed to qualify for a 1% increase in the Medicaid match rate if they offered preventive services with no cost sharing. Finally, on January 1, 2014, enrollment under Medicaid expansion became active. In a study of breast and colorectal cancer screening under the ACA, Fedewa et al. divided states occupying these time periods into very early adopters (5 states and the District of Columbia, 2010–2011); early expansion states (21 states, 2012–2014); late expansion states (7 states, 2015 to present); and non-expansion states (12 states presently) [7]. Setting July–September 2013 as the baseline, the Medicaid and Children’s Health Enrollment Program (CHIP) Payment and Access Commission indicated that enrollment for these two programs increased by 14 million people (24.7%) among the 49 states reporting both baseline and March 2020 data [8]. Medicaid expansion state enrollment increased by 13 million people (33.9%). Non-expansion state enrollment increased by 939,321 people (5.2%). The latter increase is attributed to the “welcome-mat effect”—previously eligible but unenrolled individuals applying for Medicaid as a result of general Medicaid outreach efforts surrounding ACA implementation.

Cancer is the third largest source of spending for Medicaid in the country, following HIV and hepatitis C [9]. Cancer is also the second leading cause of death both worldwide and in the United States, annually responsible for an estimated 9.6 million and 600,000 deaths, respectively [10]. It has long been a target of state breast, cervical, and colon cancer health programs and national public health efforts [11,12]. African American and Latina women suffer increased mortality from breast cancer at every stage compared to white women [13]. Rural Appalachians are also noted to have higher (by 15–36%) cancer mortality rates than their urban peers [14]. The ACA requires coverage of breast cancer screening and mammography, breast cancer (*BRCA*) genetic counseling and testing where indicated, and cervical cancer screening as preventive services for women [15]. It also covers colorectal cancer screening for adults aged 45 to 75 years old. Lung cancer screening for high-risk adults aged 50 to 80 and chemoprevention counseling for women at higher risk are additional covered services. Medicaid poses the opportunity to narrow the above disparities because, in expanded states, it shares in the ACA’s list of covered benefits.

A Surveillance, Epidemiology, and End Results (SEER) study of 716,364 patients with newly diagnosed cancers found a greater decrease from 2011 to 2014 in the uninsured rate in expansion (−3.0%) than non-expansion (−0.9%) states [16]. The pronounced decrease was most evident in African Americans (−3.4%), Latinos (−3.9%), and rural patients (−4.8%). This group-specific finding is supported by a population-based cancer registry study examining 40 states for the 2010–2013 and 2014 time periods [17]. A SEER-based study also comparing 2010–2013 to 2014 data found that gaps in uninsured rates between African American and white cancer patients were eliminated with traditional Medicaid expansion, with uninsured rates falling from 10.0% to 0.95% in African Americans [18]. However, the gap persisted in states using Section 1115 Medicaid waivers that provide flexibility in a state’s attempt to design and improve its Medicaid and CHIP programs. Seven states are currently using such waivers to implement their Medicaid expansion along alternative pathways [19]. This finding, particularly when considering the different cancer types included in additional studies, suggests the need for a more in-depth analysis of the benefits that cancer patients have received under Medicaid since the passage of the ACA. In this review, we will inspect the effect of Medicaid expansion on groups that have experienced the burden of cancer disparities—African Americans, Latinos, and rural populations—examining current policy, overall Medicaid impact, and impact with respect to these groups from breast and colorectal screening and genetic testing perspectives. The empirical findings will be used to suggest various healthcare reform options that could be adopted in the future.

## 2. Methods

### 2.1. Recruitment and Conduct

Following the development of a policy statement advancing cancer genomics in public health, the Policy Committee of the American Public Health Association formed a working group of members from state-level and academic public health, genetic counseling, and health services to address cancer genetic testing services and marginalized groups in light of healthcare reform. In 2020, the working group completed its study of cancer diagnostic services under the ACA and published a review of its work. Since then, the group has taken on a member from the advocacy sector and narrowed its scope to the examination of cancer genetic services in underserved groups under Medicaid expansion. Members all have either a Master’s degree or analogous graduate-level work in their respective field; three of the coauthors have an additional professional certification, such as genetic counseling, epidemiology, or health education. The current effort is based on both the original group literature review undertaken through September 2020 and the Medicaid-specialized literature review through March 2022.

### 2.2. Policy Analysis

The working group chose three main categories of literature to collect: policy-oriented (e.g., genetics and Medicaid); group-oriented (e.g., women’s health); and condition-oriented (hereditary breast and ovarian cancer (HBOC) and hereditary nonpolyposis colorectal cancer—Lynch syndrome) [20,21]. In consonance with a focus on Medicaid, breast and colorectal cancer (CRC) screening also comprise the major categories examined. The literature examined has been purposefully chosen to enable policy analysis rather than an exhaustive literature review. Inspection was based on fit within chosen categories, applicability to ongoing policy measures, and pertinence to the cancer and underserved groups theme. The original search centered on the ACA yielded 408 pieces of relevant literature, of which 29 pieces are cited in the current article. For the current search concentrating on Medicaid, an additional 149 pieces of literature—19 policy documents, 51 policy reports or briefs, 65 journal articles, and 14 news articles—were collected; 133 were examined.

## 3. Results

### 3.1. Breast Cancer Screening

Breast cancer is the second most frequently diagnosed cancer among women in the United States, and the second leading cause of death in women after lung cancer [22]. Though white women have historically displayed higher breast cancer incidence rates than African American women, these rates converged in 2012 [23]. The incidence in Latina women has remained lower than in white women, yet Latinas are more likely to be diagnosed at a younger age and more advanced stage [24].

The ACA preventive care benefits, which address breast cancer screening, are based on the U.S. Preventive Services Task Force (USPSTF) and Health Resources and Services Administration (HRSA) recommendations. Current HRSA guidelines, which define no-cost coverage for private insurance, recommend biennial screening mammography to start no earlier than age 40 and no later than age 50, and to continue through at least age 74 [25]. The USPSTF recommends biennial screening mammography for women aged 50–74 years (Table 1 and Table 2) [26,27,28,29,30].

Women at higher risk of breast cancer, as recommended by a provider, may benefit from beginning screening in their 40s [15,26]. Title I., Sec. 2713 of the ACA, dealing with quality, affordable healthcare, addresses breast cancer screening and mammography as a covered service, while Title IV., Sec. 4106, dealing with preventive services for adults eligible for Medicaid, amends the Social Security Act to include clinical preventive services that are assigned a grade of A or B by the USPSTF [4]. These ratings apply to services where high certainty exists that the net benefit is substantial to moderate. Women on Medicaid in expansion states are entitled to the same screening and preventive services as those who are covered by private insurance or in group plans. However, women who qualify for Medicaid based on other traditional eligibility pathways are considered “optional” under Medicaid, with the scope of coverage determined by the state [25].

Prior to the ACA, most states required copayments for preventive services from adult Medicaid enrollees. Cost-sharing was typically low, but was on the rise in some states. In a 2003 sample of states being examined for Medicaid copayment policies in breast and cervical cancer screening, 12 states had no copayments, 24 states required copayments, and two states waived their requirement for copayments [31]. Women with copayments for preventive services (analogous to the situation in non-expansion states today) were less likely to receive a screening mammogram than those without a copayment (OR = 0.81–0.84, 95% CI: 0.71–0.97). A study examining 2006–2008 Medicaid claims data from 44 states found that African American women were significantly less likely than white women to undergo mammography in 30% to 39% of the states analyzed (OR = 0.85 in 13 states, 95% CI: 0.60–0.93) [32]. Latina (OR = 1.43 in 24 states, 95% CI: 1.08–2.04) and Asian American (OR = 1.32 in 18 states. 95% CI: 1.17–2.11) women were the minority groups most likely to receive screening compared to whites.

In the post-ACA period, a 2015 survey found that six states remained with classic Medicaid, 11 states had adopted traditional Medicaid expansion, and 23 states had secured a family planning waiver or state plan amendment (alternative pathways) (10 states excluded) [33]. That same year, the Kaiser Family Foundation reported that 58% of women with Medicaid coverage had taken a mammogram in the previous two years, in contrast to only 30% of uninsured women between ages 40 and 64, and 72% of privately insured women [25]. A 2021 systematic review of 21 select articles on Medicaid expansion status by Nathan et al. indicated that 15 of the articles reported relatively higher cancer screening rates and/or earlier stage of diagnosis in expansion states, with an average increase in screening rates of 4.6% [34]. Only 2 of the 15 articles reviewed focused on breast cancer, though. A year earlier, Moss et al. published a systematic review of 48 studies, eight of which pertained to breast cancer [35]. The outlook was more critical—most of the studies concluded that Medicaid expansion was not associated with increased access to mammograms.

The first full year of Medicaid expansion has been accompanied by reports of earlier cancer diagnosis. Studies comparing 2010 to 2014 data by utilizing population-based cancer registry and SEER data detected increases in early-stage (stages I and II) diagnosis for all cancers combined of 0.8% to 9.14%, respectively, while non-expansion states showed no such increase [17,36,37]. For breast cancer specifically, the population-based registry study detected a 0.9% increase in early-stage diagnosis for expansion states, compared to 0.4% for non-expansion states, while a cancer registry study in Kentucky (which underwent expansion in 2014) found a 2.2% increase (*p* = 0.002) [17,38]. These findings must be viewed cautiously—one of the studies also looking at 2015 and 2016 data reported a decline in both magnitude and statistical significance in early-stage diagnoses, which it attributed to drainage of pent-up patient demand [36]. Ko et al., using mediation analysis of SEER data from 2010 to 2016, reported that Latina and African American women had higher odds (OR = 1.35–1.46, 95% CI: 1.30–1.42) of stage III breast cancer compared to white women [39]. Approximately half of the observed association with higher stage was explained by being uninsured or receiving Medicaid. Care must be taken in interpreting these results. Since the duration of Medicaid coverage was unavailable, the investigators combined uninsured women and those with Medicaid coverage into the same group.

A Behavioral Risk Factor Surveillance System (BRFSS) study of women aged 50–74 years found significantly lower breast cancer screening rates in non-expansion than expansion states for non-Appalachian states (2011 to 2015) (RR = 0.95, 95% CI: 0.95–0.96) [40]. Likewise, white and Latina women displayed higher screening rates in non-Appalachian expansion than non-expansion states (rate differences of at least 3.4%), though differences were minimal for African Americans in non-Appalachian states and all groups in Appalachian states. These findings suggest a continued benefit for Latina women in breast cancer screening, together with the persistence of group-specific disparities for African Americans, most prominent because rural Appalachia leads the rest of rural America in its growing African American population [41].

### 3.2. Hereditary Breast and Ovarian Cancer Genetic Testing

Hereditary breast and ovarian cancer (HBOC) associated with *BRCA1* and *BRCA2* mutations is the most common form of hereditary breast cancer. A 2015 population-based study of 396 African American women diagnosed with breast cancer before age 50 found that 12% of the study participants had *BRCA1/2* mutations, more than double what was found in white women [42].

In 2005, the USPSTF recommended that women whose family history may be associated with an increased risk for deleterious *BRCA1/2* mutations be referred for genetic counseling and evaluation for *BRCA* testing [43] (Table 1 and Table 2). Through time, the Centers for Medicare and Medicaid Services and other federal health agencies have issued clarifications broadening the criteria for coverage [44].

A case study of select large private and public payers prior to the ACA found that few payers had detailed eligibility criteria for HBOC genetic counseling [45]. Medicaid programs in Arizona, California, and New York did not cover *BRCA* testing. Illinois’ Medicaid program covered HBOC genetic counseling and testing, but lacked clearcut eligibility criteria. In Michigan, in 2008, only four of 24 health plans had written policies aligned with the USPSTF recommendations [46]. Currently, a number of states are shifting or have moved their Medicaid patients into managed care. It is to be noted that a study by Levy et al. using a national sample of 2004–2007 medical claims and insurance-related administrative data found that health maintenance organization (HMO) enrollees were significantly less likely than those enrolled in point-of-service insurance plans to receive *BRCA1/2* testing (hazard ratio (HR) = 0.73, 95% CI: 0.54–0.99) [47]. A 2005 hospital-based study at the University of Pennsylvania of 408 women with a family history of breast or ovarian cancer found, after adjustment for socioeconomic factors, that African American women were much less likely (OR = 0.28, 95% CI: 0.09–0.89) to undergo genetic counseling for *BRCA1/2* mutations than white women [48].

Facing Our Risk of Cancer Empowered (FORCE) reports that currently all but one state’s Medicaid programs cover *BRCA* genetic counseling and testing (North Carolina, initiated in mid-2021) [49,50], a vast shift from the pre-ACA environment, although some follow the Medicare model, only testing those diagnosed with cancer. Only Alabama does not cover genetic testing for hereditary cancer risk. Rhode Island’s Medicaid program focuses on *BRCA* coverage only for those in its managed care programs. A 2020 analysis of an all-payer claims database in Massachusetts noted an increase in mean monthly *BRCA1/2* tests per 100,000 Medicaid-insured women from 3.7 in 2011 to 14.7 in 2015 [51]. On average, *BRCA1/2* testing rates increased at a similar rate for both privately and Medicaid-insured women. In 2014, with the start of traditional Medicaid expansion, New York State issued criteria for coverage of *BRCA* testing in Medicaid recipients [52]. Despite the revision, a study of 3055 predominantly low-income Latina women who had undergone screening mammography between 2014 and 2016 at a Columbia University medical center in Washington Heights, New York City revealed persistent underutilization [53]. Twelve percent of the women met family history criteria for *BRCA1/2* testing, yet <5% had previously undergone testing.

In our previous piece published in 2021 [20], we outlined how the USPSTF recommendations—thus, the set of benefits offered by the ACA—leave out coverage of male breast cancer, which results in 2350 new cases and 400 deaths per year [54]. In 8828 male breast cancer patients diagnosed between 1998 and 2006 identified by the National Cancer Database, only 3.24% of cases were enrolled in Medicaid [55]. More recent data were not identified, but, given that male breast cancer, which is associated with *BRCA* mutations, is not covered by the ACA, policy revision is called for. *BRCA2* mutations have been identified in men with high-grade, aggressive prostate cancer [56], and ACA coverage of prostate cancer screening (see Table 1 and Table 2) has led to an observed 3% increase in screening among men, less than 138% of the FPL in early expansion states [57]. African Americans and Latinos shared in this increase. The National Comprehensive Cancer Network (NCCN) Genetic/Familial High-Risk Assessment Guidelines address *BRCA1/2* genetic counseling and testing criteria in at-risk males [58]. The majority of state Medicaid plans have HBOC policies that adhere to NCCN criteria, but Medicaid concordance rates in a related area, anticancer therapy, have been noted to be around 47.5%, with less concordance for African American and Latino prostate cancer treatment [59]. The truth for male *BRCA1/2* genetic counseling and testing coverage may lie in between given USPTF’s conservative stance on another test—prostate-specific antigen [60].

### 3.3. Colorectal Cancer Screening

Colorectal cancer (CRC) is the second most frequent cause of cancer-related death in the United States and globally [61]. African Americans have the highest colorectal cancer incidence among all racial-ethnic groups, with nearly 20,000 new cases identified in 2019 [62]. Though Latinos have a lower CRC incidence than whites, it is less likely to be localized at time of diagnosis. Declines in CRC incidence rates have historically occurred later in Latino than white populations [24].

The USPSTF recommends screening for colorectal cancer in all adults aged 50 to 75 years as an A recommendation and in adults aged 45–49 years as a B recommendation. The decision to offer screening to adults aged 76 to 85 years depends on the clinician [28]. Though the essential health benefits section of the ACA does not address CRC specifically, the ACA HealthCare.gov website “Preventive health services” does [15]. In accordance with USPSTF recommendations, a payer may not impose cost-sharing with respect to screening for colorectal cancer or polyp removal performed as part of a screening procedure. More frequent use of colonoscopy for high-risk surveillance is outside of the scope of the USPSTF recommendations.

Based on Medical Expenditure Panel Survey data, a nationally representative survey of the non-institutionalized population, the average annual CRC screening prevalence from the pre-ACA period, 2007–2011, was 22 per 100 adults [63]. Screening rates decreased annually for non-Latino individuals by −0.38 per 100 adults per year but remained level for Latinos. From 2012 to 2015, screening rates increased for both groups; when screening rates were averaged, the above rate converted to 0.20 per 100 adults per year for non-Latino individuals, while the rate for Latinos remained even.

In the systematic review by Moss et al., CRC screening increased in eight studies and remained the same in two studies following expansion [35]. Kentucky displayed the largest increase, 27.7%, following expansion. Two separate research teams examining 2012, 2014, and 2016 BRFSS data found CRC screening rates increasing by 7.2–8.8% in very early expansion states, 2.9–3.9% in early expansion states, 2.4–2.7% in late expansion states, and 3.8% (one study) in non-expansion states [7,64]. Both studies reported that rate changes in the very early expansion states achieved the greatest level of statistical significance. The study by Fedewa et al., which looked at both breast and colorectal cancer, attributed this statistical effect to increased insurance coverage through Medicaid early on, since having insurance is a strong predictor of CRC screening [7]. The noted difference in screening rate changes between the various Medicaid expansion periods could explain why the studies included in the systematic review did not yield the same conclusion—the investigative teams might have been looking at differing time intervals. One study performing simulation modeling on a state that has not yet expanded Medicaid—North Carolina—calculated that Medicaid expansion would have prevented 7.1–25.5 instances of CRC per 100,000 cases in African Americans, and 4.1–16.4 instances per 100,000 in white individuals [65]. In addition to the above rate comparison studies, one study examining National Cancer Database data for 2011–2012 and 2015–2016 found that Medicaid expansion was associated with an increase in stage I CRC diagnoses (*p* = 0.035) [66].

During early Medicaid expansion, various teams explored those factors predisposing to the initiation of CRC screening in new Medicaid enrollees. Two studies identified a Latino background as increasing the likelihood of screening [6,67]; two cited urban residence as a significant factor [67,68]. In the study by Zerhouni et al. looking at early expansion, expansion, and non-expansion states, Latinos experienced a 6.5% increase and African Americans an 8.1% increase in those receiving CRC screening between 2012 and 2016, though, in comparing early expansion to non-expansion states, the rise was only statistically significant for African Americans (*p* = 0.045) [64]. African Americans were found to be more likely to have undergone CRC screening than whites (OR = 1.08, 95% CI: 1.03–1.14).

### 3.4. Lynch Syndrome Genetic Testing

Hereditary nonpolyposis colorectal cancer (Lynch syndrome—LS) is the most common hereditary form of CRC. It is also a very pernicious form—polyps can progress to the cancerous state in just 30 months, compared to 10 or more years for other CRC polyps [69]. Accordingly, the NCCN High-Risk Assessment Guidelines address Lynch syndrome evaluation criteria and testing strategies [70]. Healthy People 2030 lists increasing the proportion of people with CRC who are tested for LS as a research objective and high-priority public health issue [71]. The Centers for Disease Control and Prevention consider HBOC and LS as Tier 1 conditions, applications for which the base of collected evidence on clinical validity and utility supports implementation into practice [72]. The Evaluation of Genomic Applications in Practice and Prevention (EGAPP) Working Group found sufficient evidence to recommend offering genetic testing for LS to all individuals with newly diagnosed CRC [73]. However, because they are prevention-oriented and focused on the general population, the USPSTF recommendations apply only “to asymptomatic adults 45 years or older who are at average risk of colorectal cancer,” excluding individuals who are at a high lifetime risk, such as for Lynch syndrome and familial adenomatous polyposis [28]. This omission is based on mission definition rather than level of evidence, and does not preclude the value gained from conducting LS genetic testing and screening in those at risk.

At the suggestion of FORCE, in December 2021, the USPSTF agreed to reconsider coverage of genetic testing for Lynch syndrome. FORCE reports that, nevertheless, the majority of state Medicaid programs do cover testing for LS (*MLH1*, *MSH2*, *PMS2*, or *EPCAM* mutations) [49]. Currently, six states’ Medicaid programs do not cover genetic counseling or testing for LS; limited or questionable coverage exists in two states. Two studies collected reflect the period before early Medicaid expansion. A study looking at the National Cancer Database for patients undergoing mismatch repair (MMR) deficiency testing diagnosed with CRC between 2010 and 2012 found early-stage disease to be positively associated with testing, and Medicaid, Medicare, or uninsured status to be associated with underuse of MMR deficiency testing [74]. A population-based study of 274 Louisiana Tumor Registry CRC patients from 2011 found that a medical facility’s being located in a rural area (OR = 0.49, 95% CI: 0.21–1.12) or being a public hospital (OR = 0.17, 95% CI: 0.04–0.77) is a statistically significant barrier to receiving LS genetic testing. The investigators concluded, “Low testing rates at public facilities highlight important issues in health-care delivery. Patients seen at these institutions may lack health insurance and/or hospital funding for specialized testing may be limited” [75].

Another tumor registry study, in the post-ACA period, covered samples collected between 2012 and 2016 from 767 CRC patients of diverse background in four academic medical centers [76]. Minority patients were significantly less likely (*p* = 0.02) to be referred for genetic evaluation than white patients. MMR testing rates were also lower (Latinos 3.1%; African Americans 6.0%; whites 10.7%; *p* < 0.01). African American and Latino patients were more likely than white patients to be on Medicaid or uninsured. African American race was also independently associated with a lack of referral for genetic evaluation and testing on multivariate analysis. The screening experience was quite different for 276 endometrial cancer patients evaluated for LS mutations in a large public safety-net hospital in Miami, FL between 2014 and 2016 [77]. Medical records’ immunohistochemistry (IHC, indicating likelihood of LS mutations) results were obtained for all patients treated for endometrial cancer during this time period, 79.3% of the patients being of a racial or ethnic minority background. Women of Latina ethnicity were most likely to be screened for LS (*p* = 0.006), but race did not affect the performance of screening (*p* = 0.47). In this setting, Medicaid and uninsured patients were more likely at initial treatment to be screened than patients with private insurance (*p* = 0.011).

## 4. Discussion

This review has focused on the impact of Medicaid expansion on cancer screening and genetic testing in underserved groups and the general population. The studies reviewed indicate improvements in breast cancer and colorectal cancer screening rates following the passage of the ACA, with the most marked improvements in very early expansion states. This nuance suggests that ACA policies addressed pent-up demand. Screening rates with engagement of full Medicaid expansion in 2014 continued to improve for CRC screening, but tapered for breast cancer screening according to the majority of studies. However, supported by state adoption of the U.S. Preventive Services Task Force *BRCA* testing recommendations included in the ACA, the number of Medicaid enrollees undergoing *BRCA* genetic counseling and testing continued to rise through 2015. Despite the existing need, Medicaid enrollees diagnosed with colorectal cancer have generally not experienced an increase in genetic testing for the high-risk condition Lynch syndrome. LS testing is not a part of the USPSTF recommendations, but at least one study reviewed suggests that improvements in LS testing can be brought about by thorough attention to patient necessities, as exemplified by safety-net hospitals.

Studies suggest a fixed pattern so far for racial and ethnic minorities on Medicaid who are seeking breast cancer screening. In other words, low-income Latina women have continued to benefit from the availability of mammography since before the ACA, while their African American peers have not yet so benefited. However, African Americans have experienced an increase in CRC screening, more so than whites and Latinos, at least during the early expansion period. The limited number of studies available indicate genetic testing underutilization by racial and ethnic minorities on Medicaid. Likewise, Medicaid enrollees in urban areas have benefited more from no-cost screening availability than their rural counterparts.

Medicaid coverage of cancer screening and genetic testing is complicated by an assortment of socioeconomic and psychosocial factors. Though Latina women undergoing breast cancer screening seem to have benefited from Medicaid expansion, disparities in breast cancer incidence between white and Latina women have in part been explained by lower Latina mammography utilization [24]. Cragun et al., in a Florida State Cancer Registry study of facilitators and barriers to genetic testing among breast cancer survivors aged <= 50 years, identified a lack of provider recommendation (reported by 44% of untested respondents), cost-related concerns (41%), never having heard of genetic testing (28%), and not believing that testing was necessary (18%) as diagnostic barriers among their 102 untested Latino participants [78]. The median household income in 2020 for Latino Americans was USD 20,000 less than for whites; the difference for African Americans was USD 29,000, which would contribute to differentials in ability to pay [79]. The high CRC incidence in African Americans has largely reflected differences in risk factors and healthcare access, both areas related to socioeconomic status with implications for institutional practice [80]. Our finding that African Americans needing CRC screening have benefited from expansion suggests that Medicaid has impacted individual outcomes and will likely contribute in part to the epidemiology in the future. As a whole, our findings indicate mixed improvements in cancer screening and testing for Medicaid enrollees since the passage of the ACA and suggest the need for continued policy revision (Table 3) [81,82,83,84,85,86,87,88,89,90,91].

Several studies show that safety-net hospitals have successfully provided *BRCA1/2* testing [81] and Lynch syndrome screening and testing [82] to low-income patients of diverse backgrounds. Such hospitals have a legal obligation to serve a proportionately higher number of uninsured, Medicaid, Medicare, and CHIP patients. These hospitals can serve as models for non-safety-net institutions. Services offered can include free genetic counseling, use of interpreters, written educational materials in the patient’s spoken language, and financial assistance, sometimes on the part of specially trained physicians [81]. Studies have also shown that: (1) in Medicaid expansion states, mammogram and colonoscopy use occurs more often in states with a high supply of primary care providers [92]; (2) in non-expansion states, low-income individuals needing cancer care are less likely to see a doctor due to cost issues [93]; and (3) in states moving Medicaid patients into managed care, higher physician fees are associated with greater screening for comprehensive managed care enrollees [83]. Genetic counselors are essential for the provision and interpretation of genetic tests, but such testing is often linked with screening to identify patients who may be at increased risk of cancer. Federally qualified health centers, public health departments, and physicians in private care institutions are all part of this effort. State Medicaid programs need to give deference to the levels at which physicians providing cancer screening are reimbursed.

Oklahoma and Missouri are the latest states (2021) to expand their Medicaid programs. Continued efforts should be made to get holdout states onboard. The Families First Act of 2021 increased the federal medical assistance percentage (FMAP) to states by 6.2%, though the increase, connected with the pandemic, was temporary [85]. Multiple authors have expressed the importance of increasing federal matching funds to states in connection with Medicaid reform [86,88,94]. The House-passed Incentivizing Medicaid Expansion Act of 2021, yet to be enacted, would make available to newly expanding states a 5% increase in their FMAP amount to all non-expansion enrollees (the majority in these states) [85].

State expansion efforts should be considered hand in hand with other policy prerequisites. States need to address the reimbursement of genetic counseling and testing in their Medicaid policies. In May 2021, North Carolina Medicaid issued a public solicitation for comments on the inclusion of *BRCA* counseling and testing, following which *BRCA* criteria became effective in state Medicaid policy on July 1, 2021 [50]. Such activities need to continue to be replicated in other states. Advocacy efforts through organizations such as FORCE, the American Cancer Society’s Cancer Action Network, and Lynch Syndrome International continue to influence the interpretation of the ACA requirements and the adoption of state-level coverage. In anticipation of changing their coverage policies, states need to seriously consider assessment of the availability and distribution of genetic counselors within their territory.

States have engaged in wholesale efforts to increase flexibility and savings in their Medicaid programs. In January 2021, Tennessee obtained a block grant waiver for its Medicaid program [95]. This move allows the state unprecedented flexibility to decide who is covered and what services it pays for. For a much longer time, states such as California, North Carolina, and Rhode Island have shifted the bulk of their Medicaid enrollees to managed care. Concerns over block grants revolve around administrative barriers to new enrollees and denial of access to services such as prescription drugs for serious and costly illnesses such as cancer and hepatitis [88,95]. Managed care concerns center on delayed care and services for those persons not yet switched over (20% of Medicaid enrollees in the case of Medi-Cal; 10% in the case of Rhode Island’s Neighborhood Health Plan), who may be ineligible for *BRCA* testing coverage [49]. As a service provider within the Medi-Cal system, though, Kaiser Permanente rated in the 95th percentile on National Committee for Quality Assurance measures for breast and cervical cancer screening [96].

Limitations: The subject matter of this review did not include triple-negative breast cancer and ovarian cancer. In our analysis we did not perform a general state-by-state examination of Medicaid screening and genetic testing policies, an ongoing effort of the National Coordinating Center for the Regional Genetics Networks (NCC) [97]. The authors themselves are geographically separated, a process limitation in comparing literature.

## 5. Conclusion: An Uneven Expansion and Filling

In assessing the existing literature on Medicaid, cancer screening, and genetic testing, this review has supported some expected trends, e.g., improvement in breast and colorectal cancer screening rates in general following the ACA’s passage, and shown a more heterogeneous set of findings for racial and ethnic minorities than might have been expected without review. Urban centers continue to benefit from cancer screening services more than rural areas. The ACA has led to a widening of state Medicaid programs adopting criteria for the coverage of cancer genetic testing, but such improvements have not touched all groups and not all states have shifted from traditional Medicaid. Individual and collective advocacy, institutional, and governmental means are at hand to realize the equitable expansion of Medicaid. The public health goal of protecting and promoting the health of all people in all communities can yet come to pass in the cancer genetic testing and screening space.

## Figures and Tables

**Table 1:** Abridged Affordable Care Act (ACA) and U.S. Preventive Service Task Force (USPSTF) policies relating to cancer coverage *.

Condition	Affordable Care Act (ACA) P.L. 111–148 [4] Statement (Abridged)	Related U.S. Preventive Service Task Force (USPSTF) Recommendations (Condensed)
Breast and Ovarian Cancer	SEC. 2713 (a). A group plan or health insurance issuer must not impose cost-sharing requirements for evidence-based items or services that have an A or B rating from the USPSTF; or with respect to women, are provided for in HRSA comprehensive guidelines; or that fit with USPSTF recommendations regarding breast cancer screening, mammography, and prevention	The U.S. Preventive Services Task Force recommends biennial screening mammography for women aged 50 to 74 years. Women at higher risk may benefit from beginning screening in their 40s [26]. Screen women who have family members with breast, ovarian, tubal, or peritoneal cancer or who have an ancestry associated with *BRCA1/2* gene mutations with an appropriate brief familial risk assessment tool. Women who are positive should receive genetic counseling and, if indicated, genetic testing [27]. This recommendation applies to women who are asymptomatic for *BRCA*-related cancer [27].
Colorectal Cancer	SEC. 2713 (a). A group plan or health insurance issuer must not impose cost-sharing requirements for evidence-based items or services that have an A or B rating from the USPSTF	Screen for colorectal cancer in all adults age 50 to 75 years (A recommendation) and age 45 to 49 years (B recommendation). The risks and benefits of different screening methods vary [28]. These recommendations apply only to asymptomatic adults 45 years or older who are at average risk of colorectal cancer, excluding individuals who are at a high lifetime risk, such as for Lynch syndrome and familial adenomatous polyposis [28].
Prostate Cancer	SEC. 4106. ELIGIBLE ADULTS IN MEDICAID. Section 1905 (a) of the Social Security Act is amended to read: other diagnostic, screening, preventive services, including any clinical preventive services that are assigned a grade of A or B by the USPSTF	The decision to undergo periodic PSA-based screening for prostate cancer should be an individual one. Men should discuss the potential benefits and harms, and their values and preferences, with their clinician [29]. This recommendation does not include the use of the PSA test for surveillance after diagnosis or treatment of prostate cancer and does not consider PSA-based testing in men with known BRCA gene mutations who may be at increased risk for prostate cancer [30]. Based on the available evidence, the USPSTF is not able to make a separate, specific recommendation on PSA-based screening for prostate cancer in African American men … [or] men with a family history of prostate cancer [29].

* Adapted from *Journal of Cancer Policy*, *28,* Modell, S.M.; Allen, C.G.; Ponte, A.; Marcus, G. Cancer genetic testing in marginalized groups during an era of evolving healthcare reform. 100275, Copyright Elsevier, **2021** [20].

**Table 2:** ACA HealthCare.gov preventive services description and interventions relating to cancer coverage *.

Condition	ACA HealthCare.Gov Website [15] Preventive Services Description	ACA HealthCare.Gov Website [15] Interventions Covered
Breast and Ovarian Cancer	The Affordable Care Act covers mammograms for women over age 50 to 74; and requires health insurance plans to cover these services for women at higher risk of breast cancer: Counseling about BRCA genetic testing Counseling about breast cancer chemoprevention	For women only: Screening mammography *BRCA1/2* genetic counseling *BRCA1/2* genetic testing where indicated Breast cancer chemoprevention counseling
Colorectal Cancer	Under the Affordable Care Act, most insurance plans must cover screening for colorectal cancer for persons age 45 to 75. The physician helps decide which test is appropriate and how often to get screened. Some tests are done every 1 to 3 years; others every 5 to 10 years.	The ACA website does not list specific colorectal diagnostic interventions. USPSTF recommendations: Screening fecal occult blood test Screening fecal immunochemical test Screening colonoscopy Screening flexible sigmoidoscopy
Prostate Cancer	The ACA Preventive Services website does not specifically list prostate cancer. Medicaid limited benefit programs may cover PSA screening and digital rectal exams.	The ACA website does not list specific prostate cancer diagnostic interventions. USPSTF recommendation: PSA screening should be individualized.

* Adapted from *Journal of Cancer Policy*, *28*, Modell, S.M.; Allen, C.G.; Ponte, A.; Marcus, G. Cancer genetic testing in marginalized groups during an era of evolving healthcare reform. 100275, Copyright Elsevier, **2021** [20].

**Table 3:** Policy strategies and impacts.

Policy Strategy	Advantages	Disadvantages	Impact on Marginalized Groups	References
Institutional policy changes (increase culturally sensitive services, provider fees)	Enhance willingness to offer/engage in cancer diagnostic services	Increase institutional costs; specialized training required	Increase access to physicians and volume of completed tests and screens	Komenaka et al., 2016 [81]; Kidambi et al., 2016 [82]; Sabik et al., 2020 [83]
Implement statewide criteria for Medicaid coverage of cancer genetic testing	Likely first opportunity for patient to move from cancer screening to genetic testing	Need adequate number of genetic counselors	Increase genetic testing rates, leading to more precise personal management and awareness raising in family members	NC Medicaid 2021 [50]; Durst 2015 [84]
Engage more states in Medicaid expansion	Decreased rate of uninsured; earlier cancer detection	Drains state money from other fiscal targets; Reduced quality of care, e.g., in appointment availability and wait time	Reduced number of low-income and racial-ethnic minority uninsured	Cross-Call 2021 [85]; Keith 2021 [86]; Artiga et al., 2019 [87]
State Medicaid block grants	Increased flexibility according to state needs; state can benefit from shared savings	Administrative barriers to new enrollees; coverage of costly healthcare services may not be authorized	Disenrollment of low-income and racial-ethnic minorities; loss of more expensive services	Miller et al., 2021 [88]
State shift of Medicaid enrollees to managed care	Spend state dollars more efficiently; increase in preventive care	Inability to obtain cancer genetic testing in those not shifted	More patients screened for breast cancer; less *BRCA1/2* and Lynch syndrome testing	FORCE 2021 [49]; Tye et al., 2004 [89]; Phillips et al., 2000 [90]
Support advocacy efforts	Can address coverage gaps and promote new guidelines and legislation; efforts target groups in need	Requires leadership and critical number of grassroots members; need to connect with professional and legislative champions	*BRCA1/2* testing coverage for a larger variety of individuals; Lynch syndrome testing only incrementally affected	Modell et al., 2021, 2016 [20,91]

Adapted from *Journal of Cancer Policy*, *28*, Modell, S.M.; Allen, C.G.; Ponte, A.; Marcus, G. Cancer genetic testing in marginalized groups during an era of evolving healthcare reform. 100275, Copyright Elsevier, **2021** [20].

## Data Availability

No additional data is available beyond the article contents.
